# Physeal-Sparing Soft Tissue Realignment in Pediatric Patellofemoral Instability Patients: A Review of Treatment Options and Outcomes

**DOI:** 10.3390/jcm14041116

**Published:** 2025-02-09

**Authors:** Christian F. Zirbes, Alyssa Henriquez, Alaowei Amanah, Aaron D. Therien, Sebastian Perez-Espina, Emilie Dorrestein, Diana Zheng, Jason Lilly, Emily J. Luo, Michael A. Fox, Brian C. Lau

**Affiliations:** 1 School of Medicine, Duke University, Durham, NC 27710, USA; alyssa.henriquez@duke.edu (A.H.); alaowei.amanah@duke.edu (A.A.); aaron.therien@duke.edu (A.D.T.); sebastian.perez-espina@duke.edu (S.P.-E.); emily.luo@duke.edu (E.J.L.); 2 Trinity College of Arts & Sciences, Duke University, Durham, NC 27708, USA; emilie.dorrestein@duke.edu (E.D.); diana.zheng@duke.edu (D.Z.); jason.lilly@duke.edu (J.L.); 3 Department of Orthopaedic Surgery, Duke University, Durham, NC 27710, USA; michael.a.fox@duke.edu (M.A.F.); brian.lau@duke.edu (B.C.L.)

**Keywords:** patellofemoral instability, pediatrics, functional outcomes, complications

## Abstract

Patellofemoral instability is a common condition in children, with an annual incidence of approximately 50 cases per 100,000 children. Instability of the patella involves a number of structures, such as the medial patellofemoral ligament and the vastus medialis obliquus, which can be used for patellar realignment in soft tissue, physeal-sparing procedures. In this rapid review, we aim to review the surgical interventions, post-operative outcomes, and associated surgical complications of global soft tissue procedures in the management of patellofemoral instability. A search of the Medline database was conducted to identify studies evaluating the treatment and outcomes of global treatment of pediatric patellofemoral instability. The included studies analyzed the surgical management of patellofemoral instability in pediatric patients, utilizing soft tissue global procedures and reported functional outcomes, return to sport or play, and post-operative complications. A total of eight studies were included, comprising a cohort of 270 pediatric and adolescent patients and 334 knees. The average patient age was 10.6 years, with 60.4% (163/270) patients being female, and the mean follow-up duration was 58.4 months. Of the eight studies, two examined the three-in-one procedure, three examined the four-in-one procedure, one examined a combination of medial and lateral release, and two examined the Galeazzi procedure. This review underscores the variety of global physeal-sparing surgical procedures available for treating patellofemoral instability. While outcomes are generally favorable, with high rates of return to sport, recurrent residual instability and recurrent dislocation remain significant challenges, with residual instability affecting nearly half of patients. Future research should focus on exploring long-term outcomes, optimizing patient selection, and identifying the causes of recurrent instability to further enhance patient outcomes and reduce complication rates.

## 1. Introduction

A common injury among pediatric athletes, patellofemoral instability (PFI) is the partial or total dislocation of the patella and is believed to occur as a result of skeletal immaturity and a tendency to participate in high-impact activities [1]. PFI can limit the functional activity of pediatric patients and can lead to long-term injury if not properly treated. The most common type of instability is lateral instability, which is often treated with medial patellofemoral ligament (MPFL) repair or reconstruction. Skeletally immature patients generally achieve favorable outcomes following these procedures, with studies reporting good to excellent patient-reported outcomes using tools such as the Kujala [2,3,4], Tegner [5,6,7], and IKDC scores [3,8], as well as subjective knee evaluations [9,10,11,12]. In addition, rates of return to sport have also been good, with many studies reporting rates of return greater than 70%, and in some cases rates greater than 90% [4,6]. Furthermore, a majority of patients reported participation at levels equal to or greater than preoperatively [13,14,15]. However, despite these favorable outcomes, rates of recurrent dislocation following MPFL reconstruction can be high, ranging between 0 and 24.1% [2,3,5,6,16,17]. In cases of severe instability, an isolated MPFL procedure may not be adequate, necessitating a more comprehensive, global approach to treatment.

Multiple structures contribute to patellar stability, with the medial patellofemoral ligament (MPFL) and the vastus medialis obliquus (VMO) playing critical roles in maintaining lateral and medial stability [18,19,20,21]. Given the high recurrence rates of patellofemoral dislocation following isolated MPFL procedures with or without lateral release, some have proposed combination procedures involving additional knee structures [22,23]. In patients with PFI, the patella may sit laterally and require medial realignment to enhance stability. While this can be achieved with an osteotomy in adults and skeletally mature patients, such a procedure poses the risk of disrupting the growth plate in skeletally immature patients, potentially leading to limb length discrepancies [24,25]. As a result, soft tissue realignment may be the preferred approach in these cases. These combined approaches aim to address instability in a more “global” fashion, while also sparing the physes.

Although several studies have examined the outcomes of individual treatment approaches, there is no comprehensive review of these procedures in the literature. The purpose of this review is to summarize and compare global soft tissue surgical management strategies, functional outcomes, return-to-play, and complication profiles for pediatric patients with PFI. By doing so, we aim to provide both patients and clinicians with a clearer understanding of treatment options for pediatric patients with PFI, facilitating informed decision-making and optimizing long-term functional outcomes.

## 2. Materials and Methods

### 2.1. Literature Search and Screening

**Figure 1:**
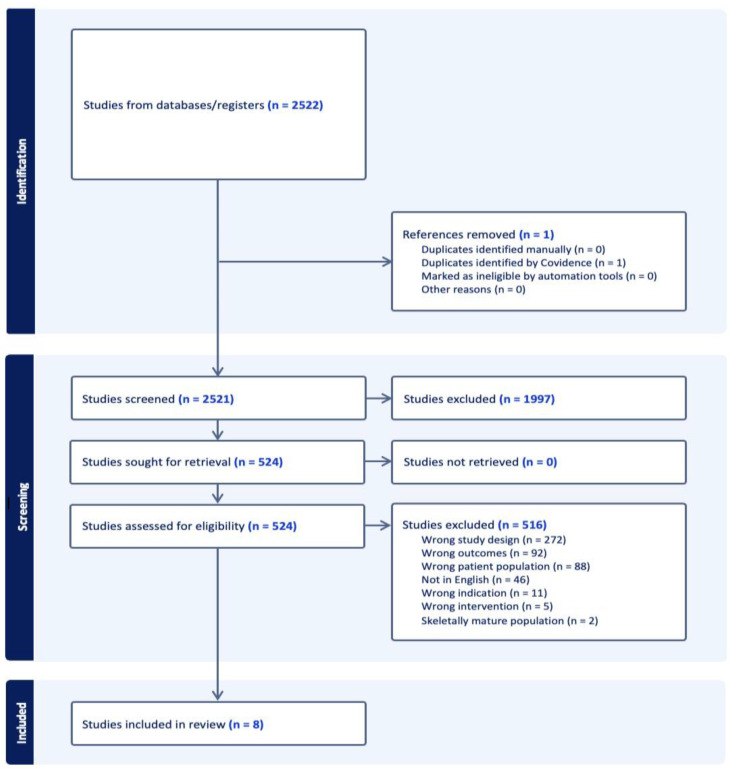
PRISMA study selection diagram.

This rapid review of the literature followed PRISMA (Preferred Reporting Items for Systematic Reviews and Meta-Analyses) guidelines where possible. The search protocol was not registered. A comprehensive search was conducted using Medline on 6 September 2024. The search was designed to identify studies evaluating the treatment and outcomes of pediatric patellar instability. The search terms targeted pediatric populations, patella joint, and instability as outlined in the detailed search strategies provided in Appendix A. The search yielded 2522 records, which were imported into Covidence (Veritas Health Innovation, Melbourne, Australia), a review management platform. One duplicate was identified and removed. The inclusion criteria were as follows: studies analyzing pediatric patients (ages 0–18 years) who were skeletally immature and underwent treatment for pediatric patellar instability with a global soft tissue procedure including management, functional score outcomes, return to sports/activities of daily living and/or complications. Case reports, review articles, cadaveric studies, non-English texts, opinion pieces, letters to the editor, and studies of adult populations were excluded. After an initial screening of titles and abstracts by three independent reviewers, 524 articles were selected for a full-text review. Of these, 8 studies met the inclusion criteria and were included in the final review (Figure 1). All voting disagreements at abstract and full-text levels were resolved in a discussion. Manual data extraction was performed by four reviewers.

### 2.2. Appraisal of Research Quality and Risk of Bias

All studies included in the current review underwent assessment for risk of bias and quality with the Methodological Index for Nonrandomized Studies (MINORS) criteria [26]. During the analysis of articles, each item in the 12-item instrument was assigned a score of 0 (not reported), 1 (inadequately reported), or 2 (adequately reported). Comparative studies could achieve a maximum score of 24, while non-comparative studies could achieve a maximum score of 16. The results of this quality and bias risk assessment are included in Table 1.

## 3. Results

### 3.1. Quality Assessment

The majority of the studies included in this review were retrospective (5/8, 62.5%), with one also having a prospective cohort. Additionally, none were comparative studies. Using the MINORS criteria [26] to assess study quality (Table 1), the average score was 11.5 (range 10–14), with most studies falling in the “fair” to “good” quality range. The most frequently assigned score was 11, indicating “fair” quality.

### 3.2. Study and Cohort Characteristics

Eight studies evaluated global PFI procedures encompassing a total of 270 patients [20,22,23,27,28,29,30,31]. Of these patients, 60.4% were female, with an average age of 10.6 years and an average follow-up of 58.4 months (Table 2). In total, three studies evaluated the four-in-one approach, two studies evaluated the three-in-one approach, and the remaining three studies addressed alternative surgical approaches to globally correct PFI. Across all studies, patients had an improvement in Kujala score of 31.6 points to 87.3 at the final follow-up, as well as an average follow-up IKDC of 72.9 (Table 3). In studies that reported on returning to activities of daily living (ADL), there was a 100% successful return rate (54/54) [20,28]. Nearly 60% of patients (21/36) reported a full return to sport at or above the level they were at preoperatively, with only 11.1% (4/36) of patients not returning to their original sport out of concern for their knee [22,23]. The overall complication rate was 32.6% (109 complications in 334 knees), with the most common complications being recurrent instability (40.2%, 37/92 knees), recurrent dislocation (8.6%, 22/255 knees), and pain (16.1%, 5/31 knees). The total subsequent operation rate was 17.7% (59 subsequent operations in 334 knees).

### 3.3. Surgical Techniques and Patient Cohort

Three-in-One Approach

**Figure 2:**
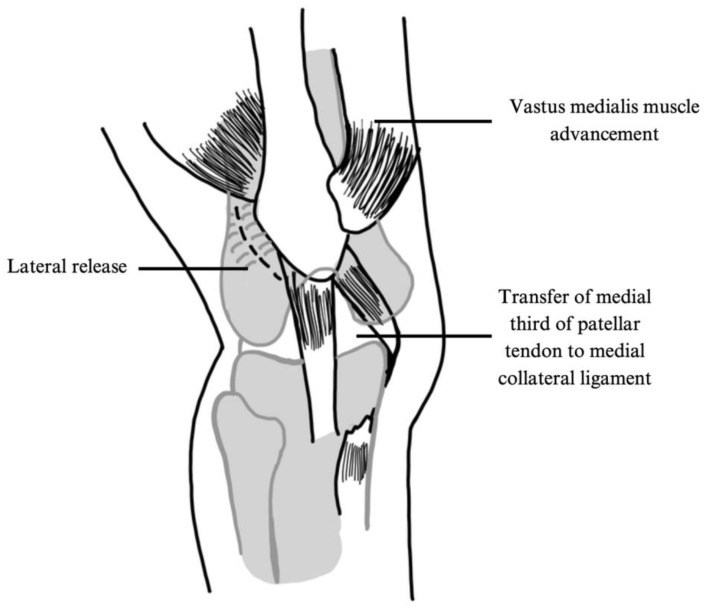
Illustration of three-in-one procedure as described by Oliva et al. 2009 [30].

A three-in-one approach typically involves lateral release, medial soft tissue realignment, and tibial tubercle realignment or patellar tendon medialization to correct patellar instability (Figure 2). The operative technique employed by both Trisolino et al. and Oliva et al. involved a lateral release, transfer of the vastus medialis obliquus, and medialization of the patellar tendon. A combined total of 151 patients and 193 knees were treated with this approach [30,31].

Four-in-One Approach

**Figure 3:**
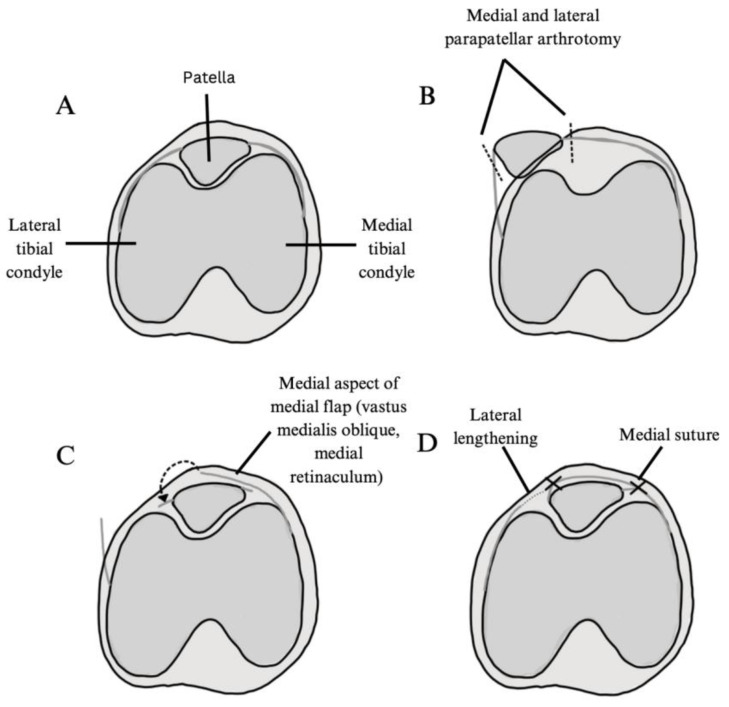
Illustration of quadricepsplasty procedure as described by Parikh et al., 2023 [23]. (**A**) Anatomical alignment. (**B**) Medial and lateral parapatellar arthrotomy (represented by dashed lines) and mobilization of the patella after wide lateral releases. (**C**) The medial aspect of the medial flap is brought over the patella (dashed arrow) and is sutured to the lateral aspect of the patella. (**D**) Medial patellar suture and lateral lengthening.

A four-in-one approach to treat PFI typically builds upon the components of the three-in-one approach with the addition of procedures to realign the knee extensor mechanism (Figure 3). Malagelada et al. specifically assessed a combination of a lateral release, medial reefing, Insall tube realignment and Roux–Goldthwait patella ligament transfer. In contrast, Danino et al. evaluated a four-part procedure consisting of a lateral release, Roux–Goldthwait ligament transfer, vastus medialis obliquus advancement, and Galeazzi procedure. Lastly, Parikh et al. utilized wide lateral releases, Insall proximal tube realignment, Roux–Goldthwait patellar tendon hemi-transfer, and stepwise quadriceps lengthening. Therefore, while all three studies utilize a lateral release, a Roux–Goldthwait procedure, and quadricepsplasty, they exhibit variation in the specific techniques employed to achieve realignment, highlighting the procedural diversity within the four-in-one approach. Across the three studies that discussed this approach, a total of 58 patients and 62 knees were evaluated [22,23,28].

Medial and Lateral Retinaculum Plasty

**Figure 4:**
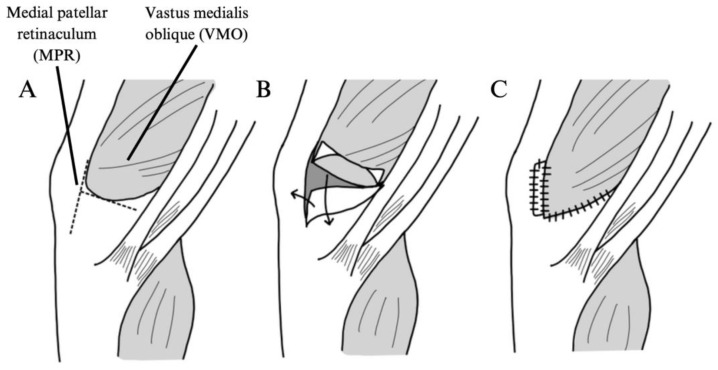
Illustration of medial retinaculum plasty as described by Li et al., 2020 [20]. (**A**) A longitudinal incision is made along the medial border of the patella, and a transverse incision is made at the junction of the vastus medialis oblique (VMO) and the medial patellar retinaculum (MPR) to the femoral condyle. (**B**) The VMO and MPR are divided, and the MPR is pulled proximally and laterally to the upper pole of the patella and fixated. The VMO is pulled distally and laterally to the medial border of the patella and fixated. (**C**) The tissues are sutured.

**Figure 5:**
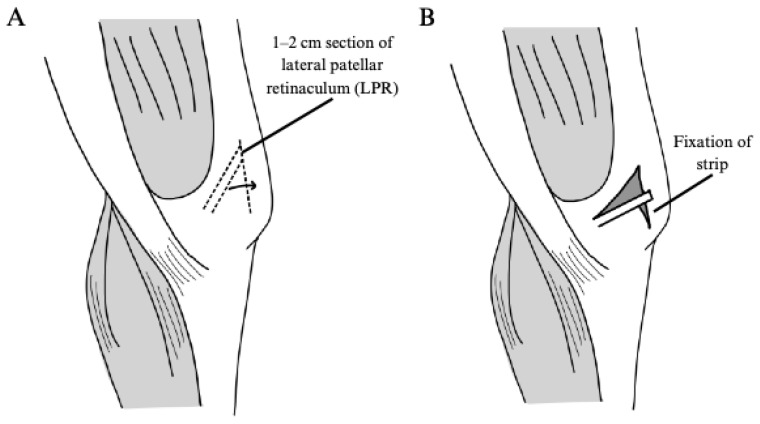
Illustration of lateral retinaculum plasty as described by Li et al., 2020 [20]. (**A**) A longitudinal incision is made along the lateral edge of the patella, and a section 1–2 cm in width is made within the lateral patellar retinaculum. (**B**) The strip is pulled to the center of the lateral patellar edge and fixated with sutures.

Li et al. examined medial and lateral retinaculum plasty as a treatment for global PFI [20]. In this technique, the vastus medialis oblique and medial patellar retinaculum are incised and fixated to the medial aspects of the patella to provide medial stabilization. Simultaneously, the lateral patellar retinaculum is incised and fixated to the lateral aspect of the patella to provide proper alignment and tension (Figure 4 and Figure 5). A total of 19 knees in 19 patients underwent a medial and lateral retinaculum plasty in the above study.

Galeazzi Procedure and Baker’s Modification

**Figure 6:**
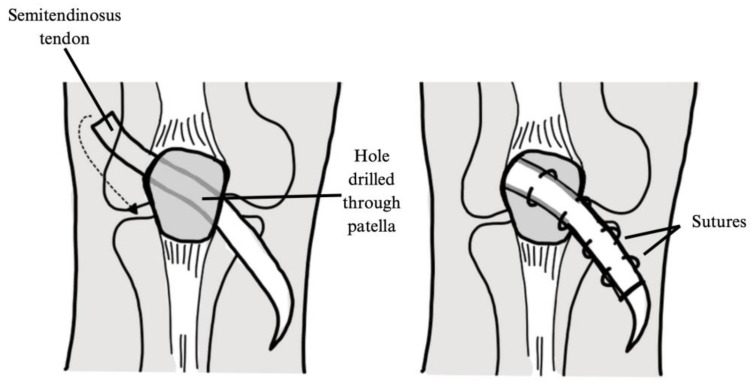
Galeazzi’s semitendinosus tenodesis as described by Grannatt et al. [29]. A hole is drilled in the patella, and the semitendinosus tendon is passed through the hole and sutured to itself.

The Galeazzi semitendinosus tenodesis was first described by Galeazzi in 1922, then modified by Fiume in 1954 and later by Baker in 1972. This procedure involves reconstructing the medial patellotibial ligament by passing the semitendinosus tendon medially to laterally through an oblique tunnel in the patella. The tendon is then sutured to itself, followed by a lateral release and medial retinacular reefing to optimize patellar alignment and stability [27] (Figure 6).

### 3.4. Patient Reported Outcomes

At final follow-up, patients demonstrated good outcomes as assessed by Kujala scores, regardless of surgical technique utilized. For the three-in-one technique, Oliva et al. reported a significant improvement from 52.4 preoperatively to 93.8 at final follow-up, a 41.4 point increase (*p* < 0.02) (Table 2). Similarly, Trisolino et al. reported a mean Kujala score of 86.4 ± 16.3, with 62.5% (85/136 knees) achieving a “good/excellent” outcome (Kujala ≥ 85) and 27.9% (38/136) having “fair” results (Kujala 65–84). For patients treated with a four-in-one technique, no preoperative scores were reported, but these 58 patients also reported a high Kujala score of 90.4 at final follow-up. Patients who underwent a medial and lateral retinaculum plasty had a significant 29.3 point improvement: from 57.6±4.2 preoperatively to 86.9 (*p* < 0.05). Patients who were treated with a modified Galeazzi procedure had lower Kujala scores at final follow-up, with Granatt et al. reporting a mean score of 79.7 [29]. However, patients still had very good outcomes, with Aulisa et al. reporting that 64.3% (9/14 knees) had an excellent outcome, and the remaining 35.7% had a good outcome [27].

Patients also reported significant improvements and good outcomes across various other outcome tools utilized. For three-in-one patients, the modified Cincinnati score significantly increased from 51.7 to 94.3 (*p* < 0.02) [30], while four-in-one patients had a high Knee Injury and Osteoarthritis Outcome Score (KOOS) at final follow-up of 93.9 ± 12.9 [23]. International Knee Documentation Committee (IKDC) scores at final follow-up were also favorable, with three-in-one patients achieving a mean score of 83.3 as reported by two studies [22,23], though Grannatt et al. reported that those undergoing a Galeazzi procedure only achieved a mean of 63.6 [29]. Finally, Li et al. reported that those undergoing a medial and lateral patellar retinaculum plasty had a significant increase in Tegner score, increasing from 2.6 ± 1.0 preoperatively to 5.0 ± 1.3 (*p* < 0.05) [20].

Overall, despite the variety of surgical techniques and patient-reported outcome tools used, patients consistently demonstrated good scores at final follow-up, with significant improvements frequently observed from preoperative values.

### 3.5. Return to Sport/Return to Activities of Daily Living

In three-in-one patients, Oliva et al. reported that less than 50% returned to sport at or above their preoperative level, with 2/24 patients (8.3%) returning at a higher level and 9/24 (37.5%) at the same level [30]. Four patients (16.7%) retired from the original sport due to concern for their knee, and instead pursued a non-weightbearing sport. Patients who underwent a four-in-one procedure demonstrated higher return-to-sport rates, with Malagelada et al. reporting 10/12 patients (83.3%) and Danino et al. reporting 20/22 knees (91%) successfully returned to sport [22,28]. Additionally, all patients in Danino et al.’s study returned to full ADLs. While no studies reported on return to sport for patients treated with a medial and lateral retinaculum plasty or modified Galeazzi procedure, Li et al. noted that all patients 19 patients who underwent retinaculum plasty returned to full ADLs [20].

### 3.6. Complications and Other Notable Findings

The rate of recurrent dislocations in patients undergoing the three-in-one procedure was 10.4% (20/193 knees), including one traumatic dislocation. Of these, 75% (15/20) required a subsequent surgery for the dislocation [30,31]. By comparison, recurrent dislocation occurred in 3.2% (2/62) of knees treated with a four-in-one procedure, both of which required surgical intervention. Recurrent dislocation was not explicitly reported in studies on retinaculum plasty or the Galeazzi procedure.

Recurrent instability was the most common complication reported in those undergoing the four-in-one procedure, affecting 15.5% (9/58) of knees, 55.6% (5/9) of which required corrective surgery [23,28]. Patients treated with a modified Galeazzi procedure had an even higher rate of recurrent instability, with 82.4% (28/34) of knees in a single study suffering from this complication, with 42.9% (12/28) requiring surgery for correction [29]. Recurrence of instability was not explicitly mentioned in the three-in-one studies or in the retinaculum plasty study.

Additional surgeries included 22 of 168 knees (13.1%) in Trisolino et al.’s study of three-in-one procedure patients undergoing a subsequent staged surgical procedure [31], and two out of the three reported cases of subluxation (3/46 knees, 6.5%) in four-in-one patients also required surgical correction [28].

Other notable complications in three-in-one patients included a single report of avascular necrosis of the patella [31]. In four-in-one patients, complications included minor patellar maltracking treated with therapy (3/16 knees, 18.8%) [22], pain (2/16 knees, 12.5%) [22], and superficial wound infections (4/70 knees, 5.7%) treated with either local wound care or antibiotics [22,23,28]. For retinaculum plasty patients, knee pain was the most common complication, occurring in 3/19 patients (15.8%) [20]. Other complications included loss of flexion (less than 10°) and a lateral patellar shift greater than 1.5 cm, both occurring in two patients (10.5%). Finally, complications reported in patients treated with a Galeazzi procedure include transient saphenous nerve defect (4/48 knees, 8.3%), superficial wound infection (1/48, 2.1%), and mild wound dehiscence (1/48, 2.1%).

## 4. Discussion

This review demonstrates that surgical treatment of pediatric patients with a global approach generally results in good to excellent outcomes at final follow-up, regardless of the technique employed. Across eight studies of fair to good quality, patients achieved an average follow-up Kujala score of 87.3, reflecting a 31.6-point increase, and a follow-up IKDC of 72.9. Most patients were able to return to their original sport, with 21/36 returning at a level equal to or greater than their preoperative performance. Only a minority (4/36, 11.1%) of patients changed to a non-weightbearing sport out of concern for their knee.

However, recurrent instability remains a significant concern, affecting almost half (37/92, 40.2%) of treated knees. Of these, 46.0% (17/37) required surgery to address this issue. The rate of recurrent dislocations was comparatively low at 8.6% (22/255 knees), though a greater proportion of these knees would ultimately need surgery (17/22, 77.3%). Overall, 109 complications were reported in 334 knees for an overall complication rate of 32.6% and the total reoperation rate was 17.7% (59/334).

The techniques examined in this review were evaluated with variable outcome measures, which make it challenging to directly compare their efficacy. However, the rate of return to sport was largely similar between studies that included this statistic. The four-in-one technique produced return rates of 83% (10/12) and 91% (20/22), according to Malagelada et al. and Danino et al., respectively [22,28]. Similarly, for the three-in-one approach, Oliva et al. report a cumulative return of 83% (20/24), which suggests that this approach has similar efficacy in restoring athletic ability [30]. With regard to complications, of the studies that reported recurrent patellar instability, dislocation, or maltracking, the highest rate of instability was 83% (28/34), reported by Grannatt et al. following the Galeazzi procedure [29]. Notably, this study utilizes an oblique hole through the patella, which has previously been associated with a high rate of postoperative failures and has therefore been modified by Nietosvaara and Giordano to a longitudinal hole [32,33,34]. Comparatively, Malagelada et al. found that 25% (3/12) of patients had minor patellar maltracking after the four-in-one procedure, and Parikh et al. found that 25% (3/12) had recurrent instability after the four-in-one procedure [22,23]. This suggests that alternative techniques may be superior to the Galeazzi procedure in terms of providing long-term stability. Future studies of these respective techniques with uniform outcome measures are needed to more definitively compare their efficacy.

Though reconstruction of the MPFL is a commonly employed surgical treatment option for patients with PFI [35], patients who present with a more global laxity may have a more complex pathology that underlies their instability, and thus require additional procedures for correction which increases procedural complexity. Despite this, the outcomes in the present review aligned with those of a recent review of skeletally immature patients who underwent reconstruction of their MPFL for PFI. A review by Kalinterakis et al. comparing anatomic vs. non-anatomic techniques reported postoperative Kujala scores of 91.1 and 85.3, respectively, which were very similar to our average of 87.3 [35]. They additionally reported that 90% of patients returned to sport at a level equal to or greater than preoperatively, with less than 10% not returning. The present review found that approximately 60% of patients returned to sport at a level equal or superior to their preoperative level, markedly inferior to Kalinterakis’ findings, though the rate of patients not returning to sport was comparable. These findings suggest that, despite the potentially increased complexity posed by global instability and its associated procedures, outcomes are still favorable overall and comparable to those undergoing MPFL reconstruction.

Surgical treatment of skeletally immature patients has the added challenge of open physes that must not be disrupted lest the patient be at increased risk of limb length discrepancy. This therefore precludes the use of osseous fixation using the femur [17]. Despite this added limitation, patients treated surgically with the global instability procedures covered in this review still demonstrate excellent outcomes, at times potentially superior to skeletally mature adults undergoing MPFL reconstruction with osseous fixation. Lind et al.’s cohort of adult patients had a one-year follow-up Kujala score of 80, which was 17 points higher than their preoperative score of 63 [17]. Additionally, Hobson et al. reported a one-year follow-up IKDC of 78.6 in adults, an increase of almost 36 points from a preoperative score of 43, similar to the postoperative IKDC of 77.6 reported by Quinlan et al., also in a skeletally mature cohort [36,37]. Thus, despite the added challenge posed by skeletal immaturity, the global procedures to address instability have non-inferior functional outcome scores to those used in skeletally mature patients.

Though only a minority of patients undergoing a global procedure for instability will have a complication, the rate at which complications occur is notable, occurring in almost one in three knees (109/334, 32.6%) and two in five patients (109/270, 40.4%). The most common complication was recurrent instability, which was seen in over 40% of knees that were treated (37/92), and this is particularly troubling given the initial indication for the surgical procedure. This rate varied by procedure, ranging from as high as 82.4% (28/34) in a single study that utilized the Galeazzi procedure [29] to as low as 15.5% (9/58) across two studies reporting on the four-in-one procedure [23,28]. Of the knees that had recurrent instability, 17 needed a subsequent operation to address this issue, resulting in a reoperation rate of 46.0% in those with instability and an overall reoperation rate for instability of 18.5% (17/92 knees).

Importantly, the overall rate of recurrent dislocation was 8.6%, occurring in only 22/255 knees. Despite this low overall rate, in those who experienced recurrent dislocation, 77.3% (17/22) of the knees needed a surgical intervention, or 6.7% (17/255) overall. Similarly to recurrent instability, these rates varied by procedure: 10.4% (20/193) in three-in-one procedures, and 3.2% in a two studies utilizing the four-in-one procedure [1,22]. Recurrent dislocation was not explicitly mentioned to be present or absent in patients who had undergone a medial and lateral retinaculum plasty or the Galeazzi procedure.

When compared to skeletally immature patients undergoing a MPFL reconstruction, the overall complication rate is higher for patients undergoing a global instability procedure: 40.4% versus 24.3% of patients [35], which may be reflective of the increased complexity posed by global instability and the surgical treatment options. Additionally, the complication profile is different, with Kalinterakis et al. reporting that the most common complications were subluxation (6.9%) and recurrent dislocation (5.9%), with a subsequent operation rate of 4.6%. Not only did our primary complication of recurrent instability occur at a rate of 40.2%, but recurrent dislocation was almost one and a half times more common at 8.6%. Finally, our subsequent operation rate was 17.7%, further lending credence to the complexity of global procedures and treating patients with global instability.

### Strengths and Limitations

This review is the first to our knowledge to provide a comprehensive analysis of surgical outcomes in pediatric patients with patellofemoral instability across multiple global soft tissue techniques. However, several limitations must be acknowledged. Many of these limitations stem from inherent biases within the original studies included in this review, despite efforts to mitigate them through our assessment of study quality and risk of bias. The majority of the studies reviewed were retrospective and rated as fair to good quality by MINORS criteria, and there were no comparative studies. Significant heterogeneity across studies—particularly in patient characteristics (e.g., age, number of prior dislocation episodes, preoperative activity levels), functional outcome measures, and surgical techniques, even within procedural groupings—further complicates interpretation. Data extraction and MINORS grading were conducted by four authors (SPE, ED, DZ, JL), raising potential concerns about inter-rater reliability. To address this, we conducted a preparatory meeting before article screening and extraction to establish and clarify guidelines and objectives. Finally, it is impossible to account for all patient-specific factors, and as such, treatment decisions must ultimately rely on the clinician’s expertise and individualized patient care.

## 5. Conclusions

This review underscores the variety of global physeal-sparing surgical procedures available for treating patellofemoral instability. While outcomes are generally favorable, with high rates of return to sport, recurrent residual instability and recurrent dislocation remain significant challenges, with residual instability affecting nearly half of patients. Future research should focus on exploring long-term outcomes, optimizing patient selection, and identifying the causes of recurrent instability to further enhance patient outcomes and reduce complication rates.

## Figures and Tables

**Table 1:** Risk of bias assessment.

Author, Year	Title	Risk of Bias Assessment (MINORS Score)
Aulisa, 2012 [27]	Galeazzi’s modified technique for recurrent patella dislocation in skeletally immature patients	13
Danino, 2020 [28]	Four-in-one Extensor Realignment for the Treatment of Obligatory or Fixed, Lateral Patellar Instability in Skeletally Immature Knee	11
Grannatt, 2012 [29]	Galeazzi semitendinosus tenodesis for patellofemoral instability in skeletally immature patients	11
Li, 2020 [20]	Combined medial and lateral patellar retinaculum plasty for skeletally immature patients with patellar dislocation and low-grade trochlear dysplasia	11
Malagelada, 2018 [22]	Results of operative 4-in-1 patella realignment in children with recurrent patella instability	12
Oliva, 2009 [30]	The 3-in-1 procedure for recurrent dislocation of the patella in skeletally immature children and adolescents	14
Parikh, 2023 [23]	4-in-1 Quadricepsplasty for Habitual and Fixed Lateral Patellar Dislocation in Children	10
Trisolino, 2023 [31]	A 20-Year Retrospective Study of Children and Adolescents Treated by the Three-in-One Procedure for Patellar Realignment	10

**Table 2:** Study cohort demographics.

Author	Study Design	Number of Subjects (n)	Number of Knees	Age, in Years (Mean ± SD, Range)	Female (n)	Follow-Up in Months (Mean ± SD, Range)	First Time or Recurrent Dislocation
Aulisa, 2012 [27]	Prospective	14	14	11.1 (9.2–13.1)	10	52.8 (49.2–69.6)	Recurrent
Danino, 2020 [28]	Retrospective	34	46	10.3 ± 2.4 (6–13)	25	51.6 ± 31.5 (12–146)	Recurrent
Grannatt 2012 [29]	Retrospective	28	34	11.1 (4.5–15.8)	19	70 (27–217)	Recurrent
Li, 2020 [20]	Retrospective	19	19	12.4 (8–15)	7	35.4 (24–48)	Both
Malagelada, 2018 [22]	Prospective	12	16	12.6 (9–16)	8	65 (36–98)	Recurrent
Oliva, 2009 [30]	Prospective	25	25	13.6 ± 3.8	7	45.6 (30–72)	Recurrent
Parikh, 2023 [23]	Retrospective	12	12	9 ± 3.8 (5–15)	7	39.3	Recurrent
Trisolino, 2023 [31]	Retrospective	126	168	11.5 ± 3.7 (4.1–17.6)	80	63.6 ± 40.8 (18–146.4)	Recurrent

**Table 3:** Cohort outcomes and complications.

Author	Kujala (Mean ± SD, Range)	IKDC (Mean ± SD, Range)	Other	Complications	Return to Sports/Activities of Daily Living (ADLs)
Aulisa, 2012 [27]			Insall: Excellent: 9/14 knees (62.5%) Good: 5/14 knees (37.5%) Poor: 0/14 knees	Transient saphenous aerve deficit: 4/14 patients (28.8%)	
Danino, 2020 [28]	Follow-up 93.0 ± 5.2 (83–100)			Recurrent instability: 6/34 patients (17.7%) Additional surgical procedure for instability: 5/34 (14.7%) patients Recurrent dislocation: 2/34 (5.9%) patients Additional surgical procedure for dislocation: 2/3 (5.9%) patients Subluxation: 3/34 (8.8%) patients Additional other surgical procedure: 3/34 (8.8%) patients Superficial wound infection: 2/34 (5.9%) patients	ADLs: 16/16 patients full function Return to Sport: 20/22 knees (91%)
Grannatt, 2012 [29]	Follow-up 79.7	Follow-up 63.6	Marx Activity Score: follow-up 8.0	Recurrent instability: 28/34 knees (82.4%) Subsequent surgery for recurrent instability: 12/34 knees (35.3) Superficial wound infection: 1/28 patients (3.6%) Mild wound dehiscence: 1/28 patients (3.6%)	
Li, 2020 [20]	Pre-op 57.6 ± 4.2 Follow-up 86.9 ± 8.1 (90–99) *p* < 0.05		Tegner: pre-op 2.6 ± 1.0 Follow-up 5.0 ± 1.3 *p* < 0.05	Knee pain: 3/19 patients (15.8%) Lateral patellar shift greater than 1.5cm: 2/19 patients (10.5%) Loss of flexion < 10°: 2/19 patients (10.5%)	ADLS: 19/19 patients full function
Malagelada, 2018 [22]	Follow-up 83.4 ± 11.5	Follow-up 79.5 ± 12.6		Minor patellar maltracking treated with therapy: 3/12 patients (25%) Pain: 2/12 patients (16.7%) Keloid scar formation: 1/12 patients (8.3%) Superficial wound infection treated with antibiotics: 1/12 patients (8.3%) Recurrent Dislocation: 0 patients	Return to sport: 10/12 patients (83.3%)
Oliva, 2009 [30]	Pre-op 54.4 Follow-up 93.8 *p* < 0.02		Insall Salvati: Pre-op 1.0 Follow-up 1.0 *p* = ns Cincinnati: Pre-op 51.7 ± 12.6 Follow-up 94.3 ± 10.8 *p* < 0.02	Traumatic dislocation: 1/25 patients (4%) Subsequent surgery for dislocation: 1/25 patients (4%) Lost to follow-up: 1/25 patients (4%)	Return to sport: Higher than preoperatively: 2/24 patients (8.3%) Same as preoperatively: 9/24 patients 37.5%) Lower than preoperatively: 9/24 patients (37.5%) Did not return: 4/24 patients (16.7%) (due to knee in all 4 patients)
Parikh, 2023 [23]	Follow-up 90 ± 16.5	Follow-up 88.1 ± 13.8	Banff Patellar Instability: Follow-up 78.2 ± 23.1 KOOS: Follow-up 93.9 ± 12.9 HSS-Pedi FABS Activity Score: Follow-up 15.6 ± 7.5	Recurrent instability: 3/12 patients (25%) Postoperative arthrofibrosis requiring manipulation under anesthesia: 1/12 patient (8.3%) Superficial wound infection treated with local wound care: 1/12 patients (8.3%)	
Trisolino, 2023 [31]	Follow-up 86.4 ± 16.3 (range 28–100)			Lost to follow-up: 20/168 knees (11.9%) in 14/126 patients (11.1%) Recurrent dislocation: 19/168 knees (11.3%) Additional surgical procedure for dislocation: 14/168 knees (8.3%) Additional staged surgical procedure: 22/168 knees (13.1%) in 18/126 patients (14.3%) Avascular necrosis of the patella: 1/126 patients (0.8%)	

ADL—activity of daily living; Cincinnati—Cincinnati Knee Rating System; HSS-Pedi FABS—Hospital for Special Surgery Pediatric Functional Activity Brief Scale; IKDC—International Knee Documentation Committee Subjective Knee Form; Insall—Insall Knee Rating System; KOOS—Knee Injury and Osteoarthritis Outcome Score; Pre-Op—pre-operative; Tegner—Tegner Activity Scale.

## Abbreviations

The following abbreviations are used in this manuscript:

MPFL	Medial patellofemoral ligament
PFI	Patellofemoral instability
MINORS	Methodological Index for Nonrandomized Studies
IKDC	International Knee Documentation Committee Subjective Knee Form
ADL	Activities of daily living
AKPS	Anterior knee pain scale
HSS-Pedi FABS	The Hospital for Special Surgery Pediatric Functional Activity Brief Scale
KOOS	Knee Injury and Osteoarthritis Outcome Score
Pre-Op	Preoperative

## Appendix A

**Table A1:** Medline search strategy.

Set #	Search Strategy	Results
1. Pediatrics	“Adolescent”[Mesh] OR “Child”[Mesh] OR “Child, Preschool”[Mesh] OR “Hospitals, Pediatric”[Mesh] OR “Infant”[Mesh] OR “Infant, Newborn”[Mesh] OR “Neonatology”[Mesh] OR “Minors”[Mesh] OR “Pediatrics”[Mesh] OR “Pediatric Anesthesia”[Mesh] OR “Pediatric Emergency Medicine”[Mesh] OR “Perinatology”[Mesh] OR “Puberty”[Mesh] OR adolescent[tiab] OR adolescents[tiab] OR adolescence[tiab] OR baby[tiab] OR babies[tiab] OR boy[tiab] OR boys[tiab] OR boyhood[tiab] OR child[tiab] OR childhood[tiab] OR children[tiab] OR “emerging adult”[tiab] OR “emerging adults”[tiab] OR girl[tiab] OR girls[tiab] OR girlhood[tiab] OR infant[tiab] OR infants[tiab] OR infancy[tiab] OR juvenile[tiab] OR juveniles[tiab] OR kid[tiab] OR kids[tiab] OR minors[tiab] OR newborn[tiab] OR newborns[tiab] OR neonatal[tiab] OR neonate[tiab] OR neonates[tiab] OR neonatology[tiab] OR neonatologist[tiab] OR neonatologists[tiab] OR preterm[tiab] OR prematurity[tiab] OR preadolescent[tiab] OR preadolescents[tiab] OR preadolescence[tiab] OR puberty[tiab] OR pubescent[tiab] OR pubescence[tiab] OR prepubescent[tiab] OR prepubescence[tiab] OR pediatric[tiab] OR pediatrics[tiab] OR paediatric[tiab] OR paediatrics[tiab] OR PICU[tiab] OR Pediatrician[tiab] OR pediatricians[tiab] OR paediatrician[tiab] OR paediatricians[tiab] OR pediatric[tiab] OR pediatrics[tiab] OR paediatric[tiab] OR paediatrics[tiab] OR stepchild[tiab] OR stepchildren[tiab] OR schoolchild[tiab] OR schoolgirl[tiab] OR schoolgirls[tiab] OR schoolboy[tiab] OR schoolboys[tiab] OR “school age”[tiab] OR “school aged”[tiab] OR toddler[tiab] OR toddlers[tiab] OR teen[tiab] OR teens[tiab] OR teenager[tiab] OR teenagers[tiab] OR teenaged[tiab] OR teenage[tiab] OR youth[tiab] OR youths[tiab] OR youngster[tiab] OR youngsters[tiab] OR “young person”[tiab] OR “young persons”[tiab] OR “young people”[tiab]	5,024,019
2. Patella	“Patellofemoral Joint”[Mesh] OR “Patella”[Mesh] OR Patell*[tiab]	31,417
3. Instability	“Joint Instability”[Mesh] OR “Joint Dislocations”[Mesh] OR “Patellar Dislocation”[Mesh] OR sublux*[tiab] OR disloca*[tiab] OR instabil*[tiab] OR unstabl*[tiab]	334,717
4. COMBINE	#1 AND #2 AND #3	2522

## References

[1] Tan S.H.S., Sin Q.S., Tan L.Y.H., Lim A.K.S., Hui J.H. (2024). Combination of tibial tubercle transfer, medial patellofemoral ligament reconstruction, trochleoplasty and lateral release for patellofemoral instability provides good middle- to long-term outcomes in adolescents. Eur. J. Orthop. Surg. Traumatol..

[2] Abouelsoud M.M., Abdelhady A., Elshazly O. (2015). Anatomic physeal-sparing technique for medial patellofemoral ligament reconstruction in skeletally immature patients with ligamentous laxity. Eur. J. Orthop. Surg. Traumatol..

[3] Alm L., Krause M., Mull C., Frosch K.H., Akoto R. (2017). Modified adductor sling technique: A surgical therapy for patellar instability in skeletally immature patients. Knee.

[4] Kumar N., Bastrom T.P., Dennis M.M., Pennock A.T., Edmonds E.W. (2018). Adolescent Medial Patellofemoral Ligament Reconstruction: A Comparison of the Use of Autograft Versus Allograft Hamstring. Orthop. J. Sports Med..

[5] Bremond N., Prima R., Rabattu P.Y., Accadbled F., Chotel F., Konkel M., Eid A., Philippe C., Godinho A., Turati M. (2023). Isolated MPFL reconstruction with soft tissue femoral fixation technique in 54 skeletally immature patients: Clinical outcomes at 2 years follow-up. A French multicenter retrospective study. Orthop. Traumatol. Surg. Res..

[6] Husen M., Milbrandt T.A., Shah V., Krych A.J., Stuart M.J., Saris D.B.F. (2023). Medial Patellofemoral Ligament Reconstruction Using Allografts in Skeletally Immature Patients. Am. J. Sports Med..

[7] Mao Y., Li J., Li Y., Zhu J., Xiong Y., Li J. (2024). A Combined Surgical Approach for Recurrent Patellar Dislocation in Adolescents With Patella Alta and Increased Tibial Tuberosity-Trochlear Groove Distance: Improved Clinical Outcomes but Decreased Posterior Tibial Slopes in Skeletally Immature Patients at Minimum 4-Year Follow-Up. Arthroscopy.

[8] Gornick B.R., Kwan K.Z., Schlechter J.A. (2024). Medial Patellofemoral Ligament Augmentation Repair for Primary Patellar Dislocation With Concomitant Chondral or Osteochondral Injury in Children and Adolescents: Outcomes at Minimum 2-Year Follow-up. Orthop. J. Sports Med..

[9] Marsh J.S., Daigneault J.P., Sethi P., Polzhofer G.K. (2006). Treatment of recurrent patellar instability with a modification of the Roux-Goldthwait technique. J. Pediatr. Orthop..

[10] Regalado G., Lintula H., Kokki H., Kröger H., Väätäinen U., Eskelinen M. (2016). Six-year outcome after non-surgical versus surgical treatment of acute primary patellar dislocation in adolescents: A prospective randomized trial. Knee Surg. Sports Traumatol. Arthrosc..

[11] Ji G., Wang F., Zhang Y., Chen B., Ma L., Dong J. (2012). Medial patella retinaculum plasty for treatment of habitual patellar dislocation in adolescents. Int. Orthop..

[12] Zein A.M.N., Allam A.F.A., Hassan A.Z.M., Soliman A.M., Mohamed M.M.A. (2024). Outcomes of an All-Soft Tissue Fixation Technique for Reconstruction of the Medial Patellofemoral Complex Using Double-Bundle Quadriceps Tendon Autograft for Recurrent Patellar Dislocation in Skeletally Immature Patients. Orthop. J. Sports Med..

[13] Nelitz M., Dreyhaupt J., Reichel H., Woelfle J., Lippacher S. (2013). Anatomic reconstruction of the medial patellofemoral ligament in children and adolescents with open growth plates: Surgical technique and clinical outcome. Am. J. Sports Med..

[14] Örs Ç., Çaylak R., Karataş Ö., Sarpel Y. (2024). Anatomical medial patellofemoral ligament reconstruction improves sport participation and activity levels in adolescents with recurrent patellar dislocation. Jt. Dis. Relat. Surg..

[15] Rueth M.J., Koehl P., Schuh A., Goyal T., Wagner D. (2023). Return to sports and short-term follow-up of 101 cases of medial patellofemoral ligament reconstruction using gracilis tendon autograft in children and adolescents. Arch. Orthop. Trauma. Surg..

[16] Leite C.B.G., Hinckel B.B., Ribeiro G.F., Giglio P.N., Santos T.P., Bonadio M.B., Arendt E., Gobbi R.G. (2023). Medial patellofemoral ligament reconstruction in skeletally immature patients without correction of bony risk factors leads to acceptable outcomes but higher failure rates. J. ISAKOS.

[17] Lind M., Enderlein D., Nielsen T., Christiansen S.E., Faunø P. (2016). Clinical outcome after reconstruction of the medial patellofemoral ligament in paediatric patients with recurrent patella instability. Knee Surg. Sports Traumatol. Arthrosc..

[18] Monaco E., Criseo N., Annibaldi A., Carrozzo A., Pagnotta S.M., Cantagalli M.R., Orlandi P., Daggett M. (2023). Medial Patellofemoral Ligament Reconstruction Using Gracilis Tendon Graft and “All Suture” Knotless Anchors for Patellar Fixation. Arthrosc. Tech..

[19] Beasley L.S., Vidal A.F. (2004). Traumatic patellar dislocation in children and adolescents: Treatment update and literature review. Curr. Opin. Pediatr..

[20] Li M., Wang F., Ji G., Liu F., Fan C., Yang G., Lu J. (2020). Combined medial and lateral patellar retinaculum plasty for skeletally immature patients with patellar dislocation and low-grade trochlear dysplasia. Knee.

[21] Kucirek N.K., Lansdown D.A. (2023). Medial Patellofemoral Ligament Repair: Still a Relevant Treatment for Patellar Instability?. Oper. Tech. Sports Med..

[22] Malagelada F., Rahbek O., Sahirad C., Ramachandran M. (2018). Results of operative 4-in-1 patella realignment in children with recurrent patella instability. J. Orthop..

[23] Parikh S.N., Lopreiato N., Veerkamp M. (2023). 4-in-1 Quadricepsplasty for Habitual and Fixed Lateral Patellar Dislocation in Children. J. Pediatr. Orthop..

[24] Nelitz M., Dornacher D., Dreyhaupt J., Reichel H., Lippacher S. (2011). The relation of the distal femoral physis and the medial patellofemoral ligament. Knee Surg. Sports Traumatol. Arthrosc..

[25] Shea K.G., Styhl A.C., Jacobs J.C., Ganley T.J., Milewski M.D., Cannamela P.C., Anderson A.F., Polousky J.D. (2016). The Relationship of the Femoral Physis and the Medial Patellofemoral Ligament in Children:A Cadaveric Study. Am. J. Sports Med..

[26] Slim K., Nini E., Forestier D., Kwiatkowski F., Panis Y., Chipponi J. (2003). Methodological index for non-randomized studies (minors): Development and validation of a new instrument. ANZ J. Surg..

[27] Aulisa A.G., Falciglia F., Giordano M., Savignoni P., Guzzanti V. (2012). Galeazzi’s modified technique for recurrent patella dislocation in skeletally immature patients. J. Orthop. Sci..

[28] Danino B., Deliberato D., Abousamra O., Singh S., Klingele K. (2020). Four-in-one Extensor Realignment for the Treatment of Obligatory or Fixed, Lateral Patellar Instability in Skeletally Immature Knee. J. Pediatr. Orthop..

[29] Grannatt K., Heyworth B.E., Ogunwole O., Micheli L.J., Kocher M.S. (2012). Galeazzi semitendinosus tenodesis for patellofemoral instability in skeletally immature patients. J. Pediatr. Orthop..

[30] Oliva F., Ronga M., Longo U.G., Testa V., Capasso G., Maffulli N. (2009). The 3-in-1 procedure for recurrent dislocation of the patella in skeletally immature children and adolescents. Am. J. Sports Med..

[31] Trisolino G., Depaoli A., Gallone G., Ramella M., Olivotto E., Zarantonello P., Stallone S., Persiani V., Casadei G., Rocca G. (2023). A 20-Year Retrospective Study of Children and Adolescents Treated by the Three-in-One Procedure for Patellar Realignment. J. Clin. Med..

[32] Nietosvaara Y., Paukku R., Palmu S., Donell S.T. (2009). Acute patellar dislocation in children and adolescents. Surgical technique. J. Bone Jt. Surg. Am..

[33] Giordano M., Falciglia F., Aulisa A.G., Guzzanti V. (2012). Patellar dislocation in skeletally immature patients: Semitendinosous and gracilis augmentation for combined medial patellofemoral and medial patellotibial ligament reconstruction. Knee Surg. Sports Traumatol. Arthrosc..

[34] Schlichte L.M., Sidharthan S., Green D.W., Parikh S.N. (2019). Pediatric Management of Recurrent Patellar Instability. Sports Med. Arthrosc. Rev..

[35] Kalinterakis G., Vlastos I., Gianzina E., Dimitriadis S., Mastrantonakis K., Chronopoulos E., Yiannakopoulos C.K. (2024). MPFL Reconstruction in Skeletally Immature Patients: Comparison Between Anatomic and Non-Anatomic Femoral Fixation-Systematic Review. Children.

[36] Hobson T.E., Tomasevich K.M., Quinlan N.J., Mortensen A.J., Aoki S.K. (2022). Tape Augmentation Does Not Affect Mid-Term Outcomes of Medial Patellofemoral Ligament Reconstruction in Skeletally Mature Adolescent Patients. Arthrosc. Sports Med. Rehabil..

[37] Quinlan N.J., Tomasevich K.M., Mortensen A.J., Hobson T.E., Adeyemi T., Metz A.K., Aoki S.K. (2022). Medial Patellofemoral Ligament Reconstruction in the Pediatric Population: Skeletal Immaturity Does Not Affect Functional Outcomes but Demonstrates Increased Rate of Subsequent Knee Injury. Arthrosc. Sports Med. Rehabil..

